# The Role of Hydro-Kinesiotherapy After Intra-Articular Steroid Infiltration in the Management of Juvenile Idiopathic Arthritis: A Non-Randomized Observational Pre–Post Study with Parallel Groups

**DOI:** 10.3390/jfmk11010110

**Published:** 2026-03-06

**Authors:** Rossana Gnasso, Antonio Picone, Ayda Tavakkolifar, Stefano Palermi, Roberta Naddei, Simona Di Gennaro, Alessandro Nunzio Velotti, Mario Fusari, Tullio Alliegro, Marco Caruso, Maria Alessio

**Affiliations:** 1 Department of Public Health, University of Naples Federico II, Via Pansini 5, 80131 Naples, Italy; antonio.picone@unina.it (A.P.); tavakolifarayda@gmail.com (A.T.); stefano.palermi@unina.it (S.P.); alevelo94@gmail.com (A.N.V.); mario.fusari@unina.it (M.F.); tullio.alliegro@unina.it (T.A.); marco.caruso@unina.it (M.C.); 2 Department of Translational Medical Sciences, University of Naples Federico II, Via Pansini 5, 80131 Naples, Italy; roberta.naddei@unina.it (R.N.); simona.digennaro95@gmail.com (S.D.G.); maria.alessio@unina.it (M.A.)

**Keywords:** juvenile idiopathic arthritis, rehabilitation, intra-articular injections, hydro-kinesiotherapy

## Abstract

**Background**: Juvenile Idiopathic Arthritis (JIA) is the most prevalent rheumatological disease in childhood. It is classified into seven subtypes, each with specific clinical features. The pathogenesis of JIA involves an increased inflammatory response. Treatment options include pharmacological therapy, patient education, physical therapy, and rehabilitation. **Methods**: Patients received IAC injections and were subsequently divided into two groups: one group underwent HKT, while the other did not. The effects of HKT were assessed before treatment and one month after the IAC injections and initiation of HKT, using the Child Health Assessment Questionnaire (CHAQ), Visual Analogue Scale (VAS), and the Child Health Questionnaire—Parent Form 50 (CHQ-PF50). **Results**: Data were analyzed using the t-test. The HKT group showed non-statistically significant improvements in CHAQ and VAS scores compared to the non-HKT group. However, statistically significant differences were observed in the CHQ-PF50, particularly in the self-esteem and pain subscales. **Conclusions**: Although global differences between groups were not statistically significant, the group that underwent HKT demonstrated better scores, suggesting that HKT may reduce pain and contribute to improved quality of life in children with JIA.

## 1. Introduction

Juvenile Idiopathic Arthritis (JIA) is the most common chronic rheumatic disease in childhood and is characterized by persistent arthritis that may result in pain, joint stiffness, functional limitation, and reduced quality of life [1]. Chronic joint inflammation can impair daily activities and physical participation, highlighting the importance of interventions aimed at controlling inflammation while preserving function [2].

Intra-articular corticosteroid (IAC) injections represent a well-established therapeutic option in the management of JIA, particularly in cases of oligoarticular involvement or persistent synovitis. IAC therapy has been shown to effectively reduce local inflammation, relieve pain, and improve joint mobility, often inducing remission in the treated joint with a favorable safety profile [3,4,5,6]. For these reasons, IAC injections are widely used in clinical practice as part of a multimodal treatment approach.

Despite the clinical effectiveness of IAC injections in controlling synovitis, many children with JIA continue to experience residual functional limitations, muscle weakness, and reduced participation in daily activities following treatment [7]. Consequently, pharmacological management alone may be insufficient to fully restore function, and adjunctive rehabilitation strategies are frequently required.

Hydro-kinesiotherapy is a rehabilitation modality that may be particularly suitable in the post-injection phase. The aquatic environment reduces joint loading, facilitates movement, and may decrease pain, thereby promoting active joint mobilization and adherence to exercise in pediatric patients [8,9,10,11,12,13]. Previous studies have suggested that aquatic exercise can improve physical function and quality of life in children with JIA; however, evidence specifically addressing its role as a structured rehabilitation intervention following IAC injections remains limited.

In particular, the functional benefits of post-injection hydro-kinesiotherapy during phases of active disease have not been clearly established. This represents a relevant knowledge gap, given the widespread clinical use of both IAC injections and rehabilitation in children with JIA.

Therefore, the aim of this study was to evaluate the effects of hydro-kinesiotherapy following intra-articular corticosteroid injections in children with Juvenile Idiopathic Arthritis, focusing on functional outcomes, pain perception, and health-related quality of life.

## 2. Materials and Methods

### 2.1. Study Design

This non-randomized observational pre–post study with the parallel groups study was conducted between January 2024 and January 2025 at the Federico II University Hospital in Naples, Italy, Department of Pediatrics, Division of Pediatric Rheumatology. The Physical Medicine and Rehabilitation Department’s non-randomized observational pre–post study with parallel groups study was conducted between January 2024 and January 2025 at the Federico II University Hospital in Naples, Italy, within the Division of Pediatric Rheumatology and in collaboration with the Department of Physical Medicine and Rehabilitation.

Intra-articular corticosteroid injections were performed in the Rheumatology Department, after which patients were referred to the Physiatry Department for post-procedural evaluation and, when indicated, prescription of hydro-kinesiotherapy. The aim was to evaluate the association between intra-articular corticosteroid (IAC) injections and hydro-kinesiotherapy (HKT) in patients with Juvenile Idiopathic Arthritis (JIA), using internationally validated assessment tools, including those endorsed by the Italian Society of Pediatric Rheumatology.

### 2.2. Study Population

Patients were carefully selected to ensure homogeneity in clinical presentation, disease activity, observation time, and rehabilitation treatment. Parents of all participants provided written informed consent for both treatment and data collection.

The study sample consisted of 30 patients (25 females and 5 males), aged 3 to 17 years (mean age: 10 ± 1 years). Among them, 22 had oligoarticular JIA, 7 had polyarticular JIA, and 1 had systemic JIA. All patients presented with clinical signs of arthritis—joint swelling, positive thermotact, pain, and functional limitations. Infiltrative therapy was selected for patients with inflammation in only one joint (or in two joints in two cases) and otherwise stable disease status, with or without concurrent pharmacological treatment.

A total of 35 IAC injections were performed, as three patients required two separate joint infiltrations during the study period. Since these events occurred at different timepoints and patients had varying willingness to undergo rehabilitation therapy, each infiltration was treated as a distinct case, thus analyzed as if involving 35 separate patients.

The majority of treated joints were knees. All procedures were performed under local anesthesia, using a topical anesthetic cream (25 mg lidocaine + 25 mg prilocaine) and ice spray.

### 2.3. Intervention Treatment

#### 2.3.1. Arthrocentesis Procedure

Arthrocentesis was performed in two phases:

1. Aspiration of intra-articular effusion;
2. Intra-articular injection of triamcinolone hexacetonide, a long-acting corticosteroid.

Contraindications to arthrocentesis were strictly observed. The only absolute contraindication is the presence of periarticular infections such as cellulitis, due to the risk of introducing bacteria into the joint space. Relative contraindications include bacteremia, coagulopathies, and traumatic hemarthrosis [14].

Following the injection, patients were discharged from the day hospital unit with recommendations for:

- 48 h of complete rest.
- Ice application to the treated joint three times per day for 20 min (to reduce the risk of “micro-rock” arthritis).
- A short course of antibiotic prophylaxis with amoxicillin–clavulanic acid for five days was prescribed in accordance with local institutional protocol, particularly in younger children and in cases of repeated joint access. While routine antibiotic prophylaxis following intra-articular corticosteroid injection is not universally recommended, this precautionary approach was adopted to minimize the risk of post-procedural infection in a pediatric population.

#### 2.3.2. Study Groups

After the initial 48 h rest period, patients were divided into two groups.

**Group I** (*n* = 21, 60%) underwent hydro-kinesiotherapy (HKT):

- 5 males, 16 females
- 15 with oligoarticular JIA, 5 with polyarticular JIA, 1 with systemic JIA
- Age range: 3–16 years (mean: 10.7 years)

**Group II** (*n* = 14, 40%) did not undergo any rehabilitation:

- 1 male, 13 females
- 10 with oligoarticular JIA, 3 with polyarticular JIA
- Age range: 4–17 years (mean: 11 years)

#### 2.3.3. Hydro-Kinesiotherapy Protocol

The hydro-kinesiotherapy protocol was standardized across all participating rehabilitation facilities and delivered by physiotherapists experienced in pediatric rheumatologic rehabilitation. All therapists followed the same structured session format and therapeutic objectives. Although exercises were individually adapted to the child’s age, affected joint, and functional capacity, the overall session structure, duration, and frequency were consistent across sites. Adherence to the prescribed protocol was monitored through attendance records maintained by the rehabilitation centers. All patients included in the IKT group completed the planned treatment schedule during the observation period.

### 2.4. Outcome Measures

Patients were evaluated at two timepoints:

- **T0 (Baseline)**: Prior to IAC injection and HKT;
- **T1 (Follow-up)**: One month after IAC injection and HKT initiation.

Assessment tools included:

**a. Clinical Evaluation**

- Joint swelling;
- Thermotact (heat on palpation);
- Pain;
- Functional limitations.

**b. Functional and Quality of Life Questionnaires**

**b.1 CHAQ—Childhood Health Assessment Questionnaire** [15,16]

This validated tool assesses functional ability in children aged 6 months to 18 years. It includes 30 questions across eight domains:

- Dressing and grooming;
- Arising;
- Eating;
- Walking;
- Hygiene;
- Activities;
- Grip;
- Reach.

Responses are rated on a 0–3 scale:

- 0 = No difficulty;
- 1 = Some difficulty;
- 2 = Much difficulty;
- 3 = Unable to do;
- “Not applicable” = scored as 0.

If assistance or aids were required for a task, the domain score was adjusted to 2. The final CHAQ score is calculated as the average of domain scores, ranging from 0 to 3, with higher scores indicating greater disability.

**b.2 CHQ-PF50—Child Health Questionnaire—Parent Form 50** [17]

This questionnaire assesses both physical and psychosocial health through parental reporting. It includes the following sub-domains:

- Physical Subdomains:
- Physical functioning (PF);
- Role/social limitations due to physical health (PR);
- Bodily pain/discomfort (BP);
- General health perceptions (GH).
- Psychosocial Subdomains:
- Role/social limitations due to emotional/behavioral problems (REB);
- Self-esteem (SE);
- Mental health (HD);
- General behavior (BE);
- Parental emotional impact (PTE) and time impact (PTT);
- Family cohesion (FA).

Scores are normalized on a 0–100 scale, with higher scores indicating better health-related quality of life.

**b.3 VAS (visual analogue scale)** [18]

The VAS is a 100-mm line used to assess:

- Pain (0 = no pain; 100 = worst imaginable pain);
- Global health status (0 = excellent; 100 = very poor).

Parents were instructed to mark the line based on their child’s perceived condition. These scales were administered concurrently with the CHAQ and CHQ-PF50 at both T0 and T1.

## 3. Results

Statistical analyses were performed using the Statistical Package for the Social Sciences (SPSS), version 26.0 (IBM Corp., Armonk, NY, USA).

A two-tailed significance threshold of α = 0.05 was adopted for all statistical tests. Given the exploratory nature of the study and the limited sample size, analyses focused on within-group pre–post comparisons.

### 3.1. CHAQ—Functional Assessment

The CHAQ evaluates functional ability, particularly in terms of mobility and daily activity performance. Scores range from 0 to 3, where 0 indicates full functional capacity and 3 denotes severe functional impairment.

In the group not undergoing hydro-kinesiotherapy, a statistically significant improvement in CHAQ scores was observed between T0 and T1 (*p* = 0.011), confirming the functional benefit of intra-articular corticosteroid injections alone.

In the group undergoing hydro-kinesiotherapy, CHAQ scores also improved significantly over time (*p* = 0.0004). While the *p*-value was lower in this group, this does not indicate a greater magnitude of effect, as no effect size estimation or direct between-group comparison was performed. Consequently, the observed improvements should be interpreted as within-group changes rather than evidence of superior functional outcomes associated with hydro-kinesiotherapy.

**Figure 1:**
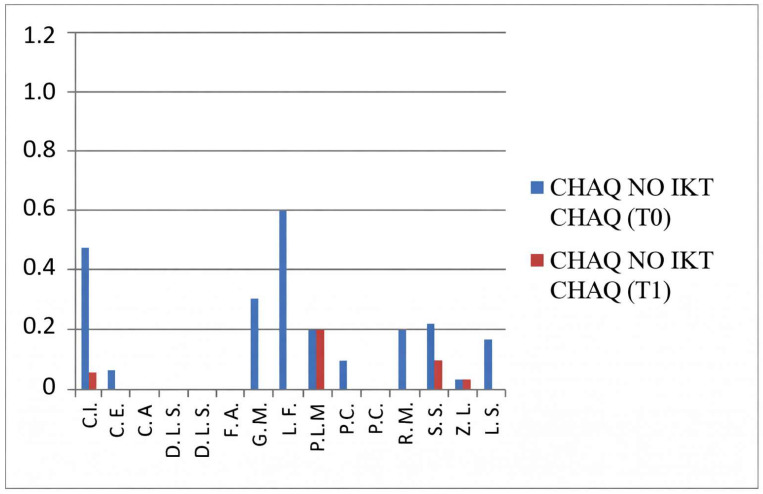
CHAQ scores at baseline (T0) and follow-up (T1) in patients not undergoing hydro-kinesiotherapy.

**Figure 2:**
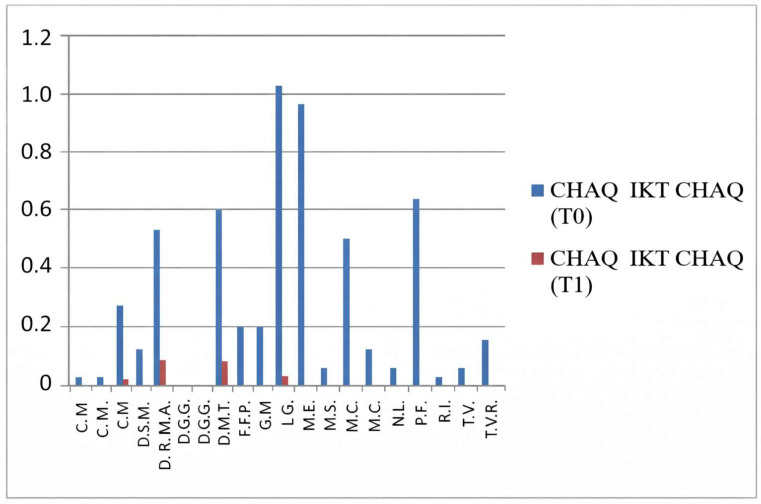
CHAQ scores at T0 and T1 in patients undergoing hydro-kinesiotherapy.

These findings suggest a potential additive or complementary association between intra-articular corticosteroid therapy and hydro-kinesiotherapy on within-group outcomes (Figure 1 and Figure 2).

These results indicate significant functional improvements over time within the group undergoing hydro-kinesiotherapy following intra-articular corticosteroid injections. As no between-group comparisons were performed, these findings should be interpreted strictly as within-group changes.

### 3.2. VAS—Pain Assessment

**Figure 3:**
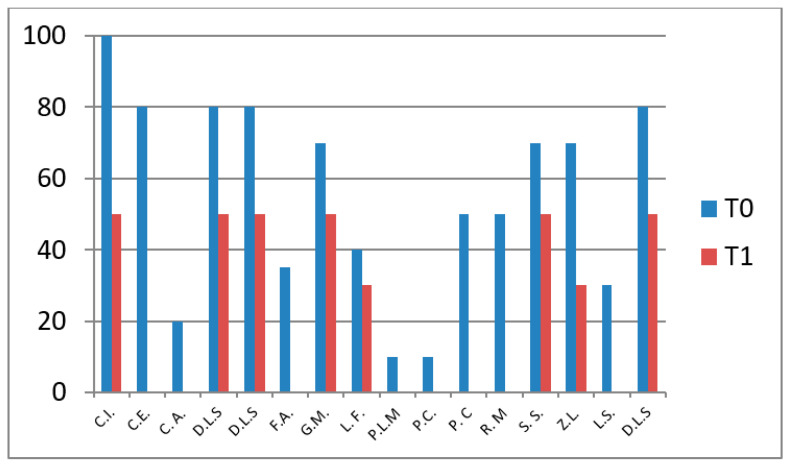
VAS pain scores at T0 and T1 in patients not undergoing hydro-kinesiotherapy.

The bar chart shows VAS pain scores at baseline (T0) and follow-up (T1) in patients who did not undergo hydro-kinesiotherapy. Most patients showed a reduction in pain intensity at T1 compared with T0, although variability between individuals was observed (Figure 3).

### 3.3. VAS—Global Assessment

**Figure 4:**
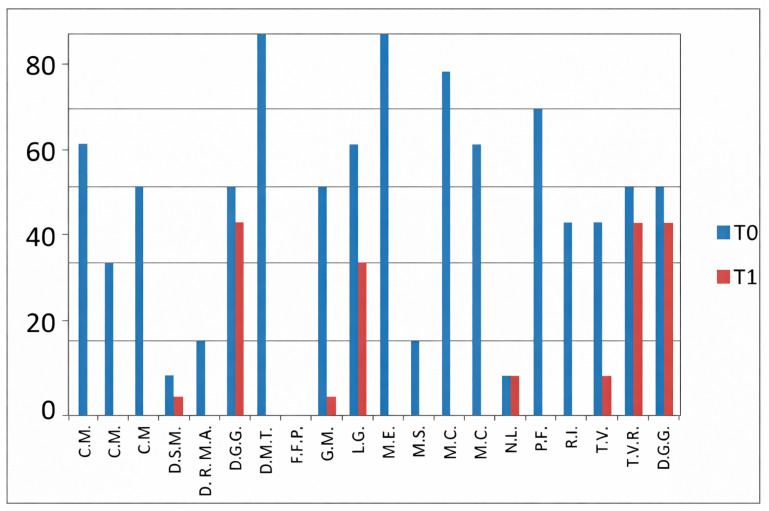
Patients’ overall subjective well-being: VAS Global Assessment.

This VAS evaluates patients’ overall subjective well-being (Figure 4).

**Figure 5:**
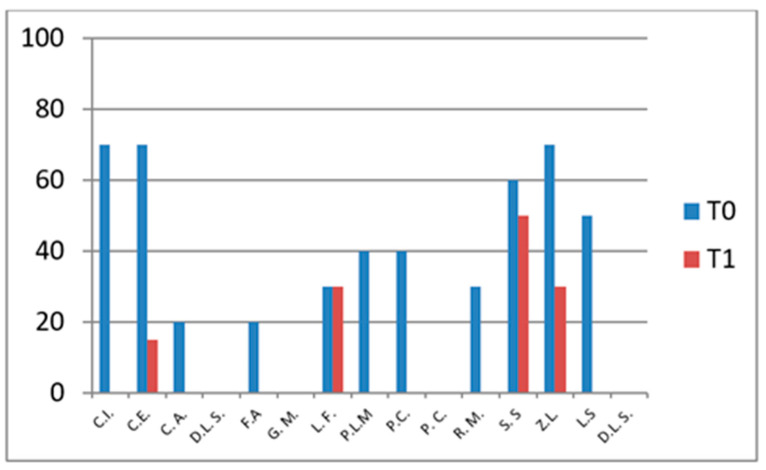
Patients who have not received HKT: VAS Global Assessment.

In the group not receiving HKT, a significant improvement was noted between T0 and T1 (*p* = 0.0005; Figure 5).

**Figure 6:**
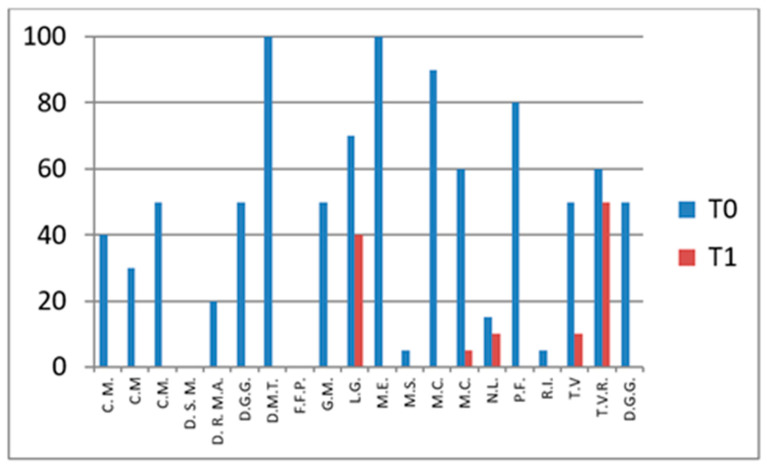
VAS global assessment scores at T0 and T1 in patients undergoing hydro-kinesiotherapy.

In the group undergoing hydro-kinesiotherapy, a significant improvement was observed between T0 and T1 (*p* = 0.0001; Figure 6). As analyses were limited to within-group comparisons, no inference regarding differential effects between treatment approaches can be made.

### 3.4. CHQ-PF50—Quality of Life Assessment

**Figure 7:**
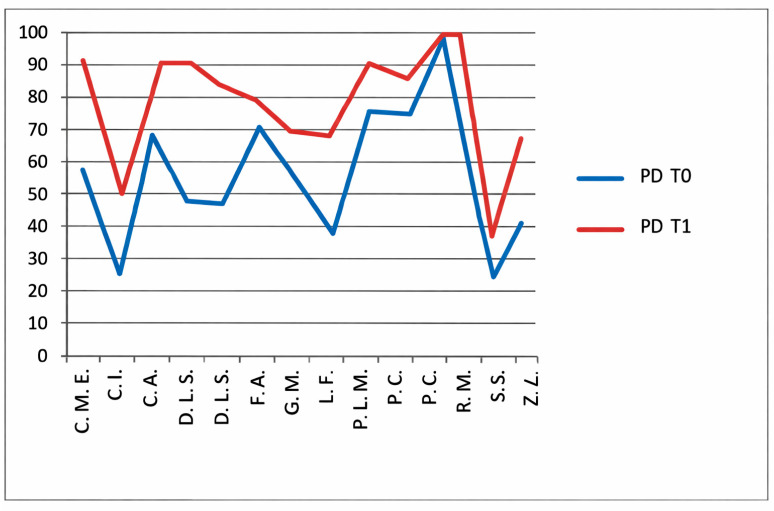
CHQ-PF50 physical health (PH) scores at baseline (T0) and follow-up (T1) in patients not undergoing hydro-kinesiotherapy.

**Figure 8:**
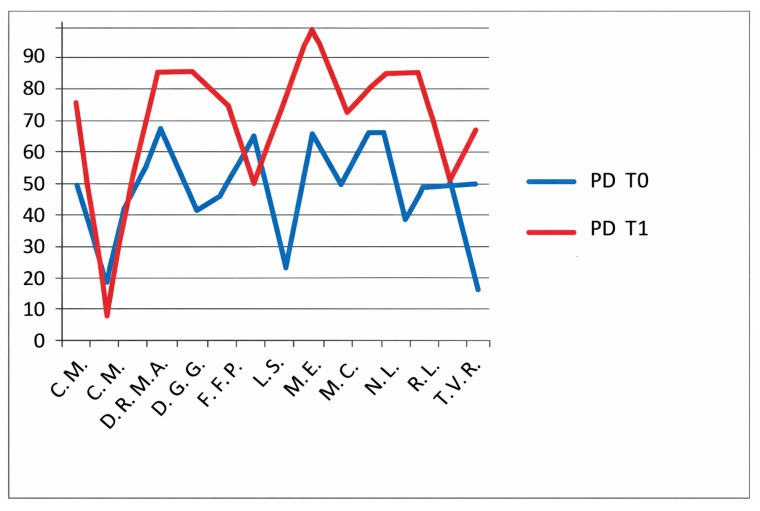
CHQ-PF50 pain domain (PD) scores T0 and T1 in patients not undergoing hydro-kinesiotherapy.

**Figure 9:**
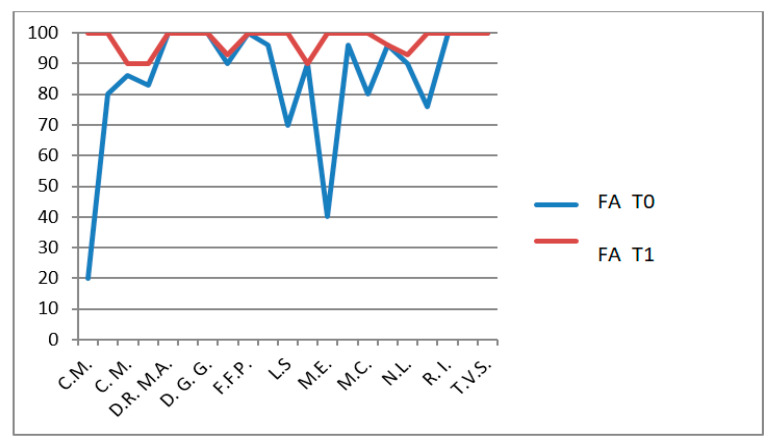
CHQ-PF50 family activities (FA) scores T0 and T1 in patients who do not undergo hydro-kinesiotherapy.

The CHQ-PF50 includes multiple physical and psychosocial subdomains, each analyzed separately. No statistical correction for multiple comparisons (e.g., Bonferroni or Holm adjustment) was applied, and therefore the risk of type I error must be considered. For this reason, all CHQ-PF50 findings should be interpreted as exploratory, intended to generate hypotheses rather than to establish definitive domain-specific effects. The CHQ-PF50, completed by parents, assesses multiple domains including physical health (Figure 7), pain (Figure 8), psychosocial functioning and family impact (Figure 9).

In the non-HKT group, significant improvements were reported in:

- Physical health (PH): *p* = 0.0034;
- Pain: *p* = 0.012;
- Family activities (FA): *p* = 0.0057.

**Figure 10:**
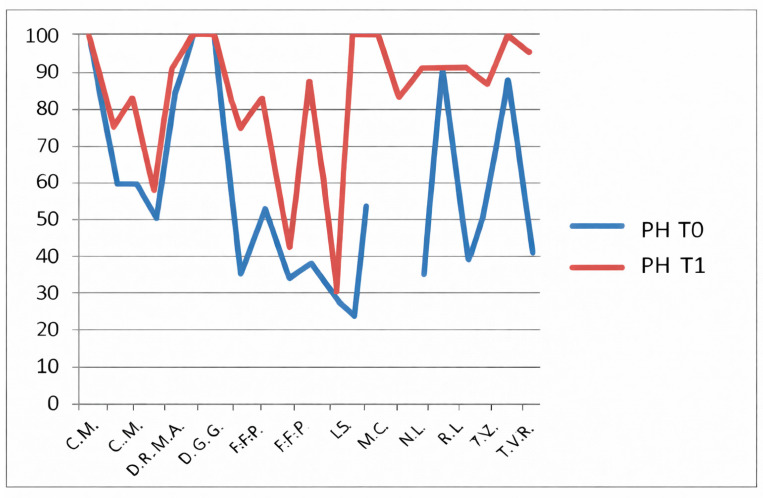
CHQ-PF50 physical health (PH) scores T0 and T1 in patients undergoing hydro-kinesiotherapy.

**Figure 11:**
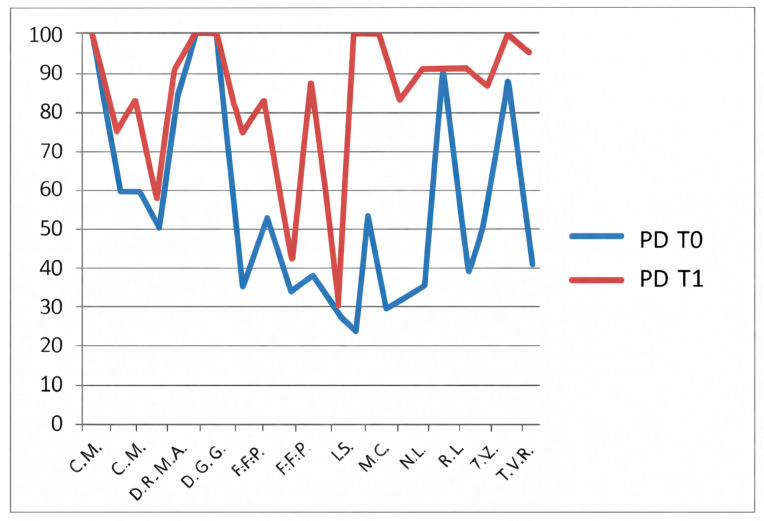
CHQ-PF50 pain domain (PD) scores at T0 and T1 in patients undergoing hydro-kinesiotherapy.

**Figure 12:**
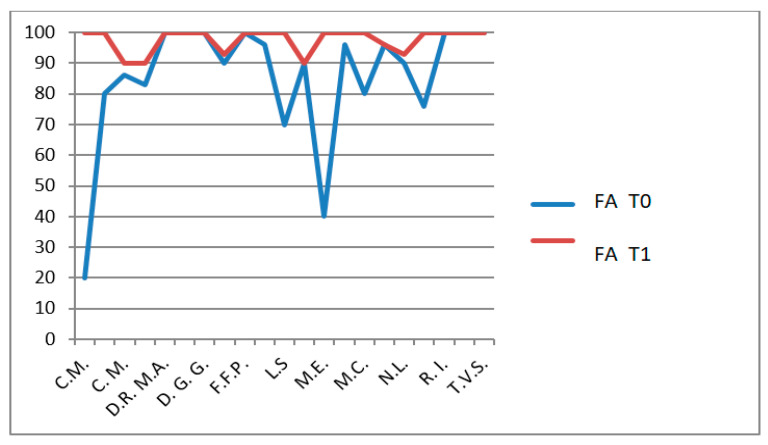
CHQ-PF50 family activities (FA) scores T0 and T1 in patients undergoing hydro-kinesiotherapy.

In the HKT group, the following improvements were observed:

- PH domain: *p* = 0.0006 (highly significant, Figure 10);
- Pain domain: *p* < 0.0001 (extremely significant, Figure 11);
- FA domain: *p* = 0.0428 (Figure 12).

**Figure 13:**
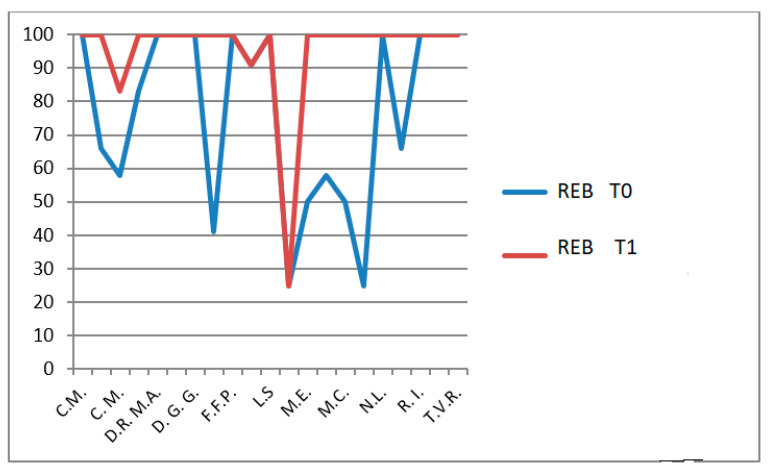
CHQ-PF50 role limitations due to emotional/behavioral problems (REB) scores at T0 and T1 in patients undergoing hydro-kinesiotherapy.

**Figure 14:**
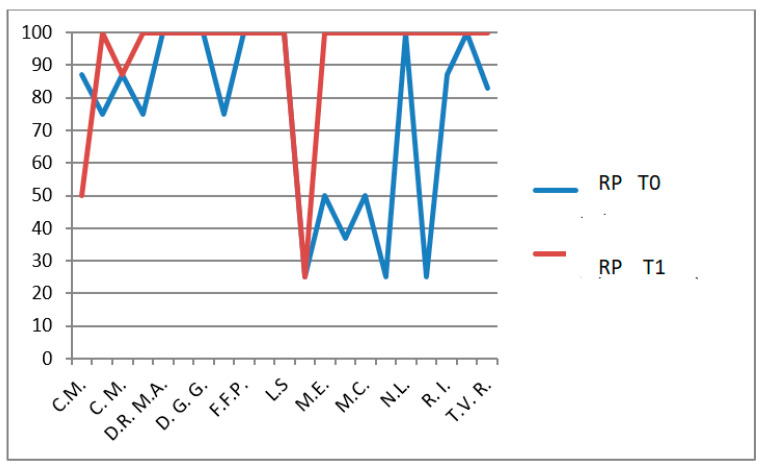
CHQ-PF50 role limitations due to physical health (RP) scores at T0 and T1 in patients undergoing hydro-kinesiotherapy.

**Figure 15:**
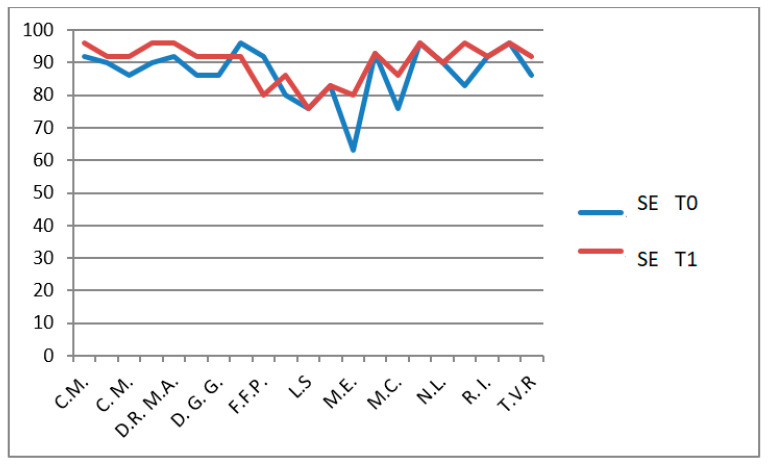
CHQ-PF50 self-esteem (SE) scores T0 and T1 in patients undergoing hydro-kinesiotherapy.

Role limitations due to emotional/behavioral problems (REB): *p* = 0.0135 (Figure 13),

- Role limitations due to physical health (REP): *p* = 0.002 (Figure 14),
- Self-esteem (SE): *p* = 0.0026 (Figure 15).

These results indicate significant within-group improvements across several quality-of-life domains following intra-articular corticosteroid treatment, with exploratory associations observed in patients who also underwent hydro-kinesiotherapy.

Taking them together, these results suggest potential associations between hydro-kinesiotherapy and selected parent-reported quality-of-life domains; however, given the explorative nature of the analyses and the absence of correction for multiple testing, these findings should be interpreted with caution.

## 4. Discussion

- In the literature, there are studies that state that aquatic therapy has been proposed as a beneficial exercise modality for children with Juvenile Idiopathic Arthritis (JIA) due to the reduced joint loading and increased freedom of movement provided by the aquatic environment. The studies by Kuntze et al. [19], Epps et al. [20] and Cavallo et al. [21], in fact, offer complementary perspectives on the effectiveness and clinical role of aquatic exercise in this population.
- Kuntze et al. investigated the effects of a structured aquatic exercise program on physical function and health-related quality of life (HRQoL) in children with JIA. Their findings suggest that aquatic exercise can lead to improvements in functional capacity and perceived well-being, supporting its use as a rehabilitation-oriented intervention focused on participation and daily activities [19].
- In contrast, Epps et al. conducted a multicenter randomized controlled trial comparing combined hydrotherapy and land-based physiotherapy with land-based physiotherapy alone, also evaluating cost-effectiveness. While both groups showed clinical improvements, no significant differences were found between interventions. These results indicate that aquatic therapy does not appear superior to land-based physiotherapy when disease activity is the primary outcome, but it remains a safe and feasible adjunct within a comprehensive rehabilitation program [20].
- Cavallo et al., through the Ottawa Panel evidence-based guidelines, synthesized available randomized trials and issued Grade A recommendations for aquatic aerobic exercise for specific outcomes, including the reduction of active joints. This work highlights that, despite modest effect sizes in individual trials, the overall body of evidence supports aquatic exercise as an effective component of structured physical activity in JIA [21].
- Differences among these studies may be partly explained by variability in intervention intensity, duration, and outcome selection. Programs with insufficient training doses or outcomes less sensitive to functional change may underestimate the benefits of aquatic therapy. Nevertheless, all three contributions consistently report good tolerability and absence of adverse effects.

The present study explores the association between hydro-kinesiotherapy and clinical outcomes following intra-articular corticosteroid injections in children with juvenile idiopathic arthritis.

The present study, in fact, confirms the effectiveness of intra-articular corticosteroid (IAC) therapy in children with Juvenile Idiopathic Arthritis, as demonstrated by significant improvements in pain, global assessment and functional outcomes in both study groups. These findings are consistent with the existing literature supporting the role of joint infiltrations in the management of localized arthritis.

- Within-group analyses showed significant improvements in CHAQ and VAS scores in both patients treated with IAC alone and those who additionally underwent hydro-kinesiotherapy. These results indicate that infiltrative therapy plays a central role in symptom control during active disease phases.

Although no statistically significant differences were demonstrated between groups for CHAQ and VAS outcomes, patients who underwent hydro-kinesiotherapy showed improvements in several CHQ-PF50 domains, particularly those related to pain perception, self-esteem, and psychosocial functioning. These exploratory findings suggest potential associations between hydro-kinesiotherapy and selected parent-reported quality-of-life domains rather than definitive functional effects.

- The aquatic environment may facilitate movement by reducing joint loading and pain, allowing children to engage more comfortably in physical activity and experience a greater sense of participation and well-being. This aspect may be particularly relevant in pediatric patients, for whom psychosocial factors play a key role in disease perception and overall quality of life.
- Several limitations must be considered. The short follow-up period may have limited the detection of longer-term rehabilitative effects, and the lack of joint-specific analyses prevents conclusions regarding differential responses by joint type. Additionally, the observational design and absence of between-group comparisons restrict causal interpretation.
- Despite these limitations, this study contributes preliminary evidence supporting the feasibility and safety of hydro-kinesiotherapy following intra-articular corticosteroid injections in children with active JIA. Further studies with larger sample sizes, longer follow-up, and joint-specific analyses are needed to better define the role of post-injection rehabilitation in this population.
- Although the majority of intra-articular corticosteroid injections were performed at the knee joint, the study did not include a systematic breakdown of outcomes by joint type. As a result, it was not possible to conduct subgroup analyses to evaluate whether the response to treatment differed between joints. Therefore, the findings should be interpreted primarily as reflecting outcomes in a cohort predominantly treated at the knee, and caution is warranted in extending these results to other joints. The intervention, in fact, improved outcomes in treated joints, predominantly the knee.
- Regarding hydro-kinesiotherapy, no statistically significant differences were observed between groups in CHAQ or VAS outcomes, as no between-group comparisons were conducted. Therefore, the present findings do not support a definitive functional superiority of hydro-kinesiotherapy over intra-articular corticosteroid injections alone. The potential role of hydro-kinesiotherapy appears to be more closely related to selected quality-of-life domains rather than objective functional measures. However, CHQ-PF50 results indicated significant improvements in self-esteem.
- First, group allocation was non-randomized, which introduces the possibility of selection bias and limits causal inference regarding the effects of hydro-kinesiotherapy.
- Second, the short follow-up duration of one month may have been insufficient to capture longer-term functional or rehabilitative effects.
- Third, neither participants nor outcome assessors were blinded, increasing the risk of expectancy and reporting bias.
- Fourth, all outcome measures were parent-reported, which may not fully reflect objective functional changes and are particularly susceptible to perception bias in the context of rehabilitation interventions.
- Additionally, some patients underwent more than one joint infiltration during the study period, and each infiltration was analyzed as an independent observation. This approach may result in statistical non-independence of observations, potentially affecting variance estimates and *p*-values.
- Finally, the lack of joint-specific analyses limits the ability to determine whether treatment effects differed by joint type. Collectively, these limitations restrict the internal validity of the study and underscore the need for randomized, adequately powered trials with longer follow-up and joint-level analyses.
- Given the observational design, non-randomized group allocation and absence of formal between-group comparisons, the present findings should be interpreted as preliminary and hypothesis-generating rather than confirmatory.

## 5. Conclusions

In conclusion, this observational study confirms the clinical benefits of intra-articular corticosteroid injections in children with Juvenile Idiopathic Arthritis and identifies exploratory within-group improvements in quality-of-life measures among patients who also participated in hydro-kinesiotherapy. Given the non-randomized design and absence of formal between-group comparisons, these findings should be interpreted as preliminary and hypothesis-generating rather than as evidence of added therapeutic efficacy. Rather than replacing land-based physiotherapy, hydro-kinesiotherapy may be particularly valuable in enhancing function, quality of life, and engagement in physical activity, especially in children with pain or significant movement limitations. No formal statistical analyses were performed to compare outcomes between groups; therefore, all reported results reflect within-group changes over time only.

## Data Availability

The original contributions presented in this study are included in the article. Further inquiries can be directed to the corresponding author.
